# Anticipated Acceptability of Blended Learning Among Lay Health Care Workers in Malawi: Qualitative Analysis Guided by the Technology Acceptance Model

**DOI:** 10.2196/62741

**Published:** 2025-04-07

**Authors:** Tiwonge E Mbeya-Munkhondya, Caroline J Meek, Mtisunge Mphande, Tapiwa A Tembo, Mike J Chitani, Milenka Jean-Baptiste, Caroline Kumbuyo, Dhrutika Vansia, Katherine R Simon, Sarah E Rutstein, Victor Mwapasa, Vivian Go, Maria H Kim, Nora E Rosenberg

**Affiliations:** 1 School of Nursing, Kamuzu University of Health Sciences, Lilongwe, Malawi; 2 RTI International, Research Triangle Park, NC, United States; 3 Baylor College of Medicine Children's Foundation, Golden Peacock, 5 Floor, Lilongwe, Malawi, +265 888 834 478; 4 Gillings School of Public Health, Chapel Hill, NC, United States; 5 Department of Pediatrics, Baylor College of Medicine, Houston, TX, United States; 6 Department of Medicine, University or North Carolina, Chapel Hill, NC, United States; 7 Department of Community and Environmental Health, Kamuzu University of Health Sciences, Blantyre, Malawi

**Keywords:** blended learning, technology acceptance model, index case testing, acceptability, partner notification, contact tracing

## Abstract

**Background:**

HIV index case testing (ICT) aims to identify people living with HIV and their contacts, engage them in HIV testing services, and link them to care. ICT implementation has faced challenges in Malawi due to limited counseling capacity among lay health care workers (HCWs). Enhancing capacity through centralized face-to-face training is logistically complex and expensive. A decentralized blended learning approach to HCW capacity-building, combining synchronous face-to-face and asynchronous digital modalities, may be an acceptable way to address this challenge.

**Objective:**

The objective of this analysis is to describe factors influencing HCW anticipated acceptability of blended learning using the Technology Acceptance Model (TAM).

**Methods:**

This formative qualitative study involved conducting 26 in-depth interviews with HCWs involved in the ICT program across 14 facilities in Machinga and Balaka, Malawi (November-December 2021). Results were analyzed thematically using TAM. Themes were grouped into factors affecting the 2 sets of TAM constructs: perceived usefulness and perceived ease of use.

**Results:**

A total of 2 factors influenced perceived usefulness. First, HCWs found the idea of self-guided digital learning appealing, as they believed it would allow for reinforcement, which would facilitate competence. They also articulated the need for opportunities to practice and receive feedback through face-to-face interactions in order to apply the digital components. In total, 5 factors influenced perceived ease of use. First, HCWs expressed a need for orientation to the digital technology given limited digital literacy. Second, they requested accessibility of devices provided by their employer, as many lacked personal devices. Third, they wished for adequate communication surrounding their training schedules, especially if they were going to be asynchronous. Fourth, they wished for support for logistical arrangements to avoid work interruptions. Finally, they wanted monetary compensation to motivate learning, a practice comparable with offsite trainings.

**Conclusions:**

A decentralized blended learning approach may be an acceptable method of enhancing ICT knowledge and skills among lay HCWs in Malawi, although a broad range of external factors need to be considered. Our next step is to integrate these findings into a blended learning package and examine perceived acceptability of the package in the context of a cluster randomized controlled trial.

## Introduction

While Malawi is making substantial progress in achieving the Joint United Nations Programme on HIV and AIDS (UNAIDS) 95-95-95 targets by 2030, the first target (HIV status awareness by persons living with HIV) has not been reached [1]. To find persons living with HIV unaware of their HIV status, the World Health Organization (WHO) promotes HIV index case testing (ICT), in which contacts of individuals living with HIV are identified, counselled, offered HIV testing, and linked to care [2]. Specifically, WHO recommends an option for voluntary assisted partner notification, in which health care workers help index clients invite their contacts for HIV testing, an approach that has been incorporated into Malawian standard of care [3-5].

Despite these recommendations, health care workers (HCWs) have limited counseling capacity that hinders full realization of voluntary assisted partner notification. For instance, formative work in Malawi identified limited counselling capacity among HCWs as a major challenge to enhancing the implementation of ICT [5]. ICT is offered by lay HCWs, cadres who lack professional training but are expected to perform select tasks within the health care system based on brief in-service training. Standard ICT training for lay HCWs is centralized, in-person, and delivered didactically. However, such approaches are logistically challenging and costly. A blended learning implementation package, which can be offered in a decentralized manner, asynchronously, and with nondidactic components is a promising alternative training approach for HCWs.

Blended learning, which combines face-to-face with digital modalities [6-8], is often more effective than digital or face-to-face training alone [9-11]. In recent years, the use of digital and blended educational approaches have increased, especially with COVID-19–driven pivots to remote learning [12-17]. Blended learning has promoted student motivation, skill-building, and achievement in several contexts [18-20]. However, blended learning has not been fully evaluated for implementation outcomes, especially among lay HCWs in low- and middle-income countries (LMICs) with limited technological literacy [21,22]. Thus, there is limited evidence as to whether lay cadres would find blended learning to be acceptable. We thus developed a cluster randomized controlled trial to assess the impact of a blended learning implementation package compared with the standard of care training on implementation, effectiveness, and cost-effectiveness outcomes [23]. Before developing this package, we conducted formative research to understand factors that may influence the acceptability of blended learning among lay HCWs in LMICs with limited technological literacy [24,25]. This formative work uses in-depth interviews (IDIs) to assess lay HCWs’ perceptions of a blended learning approach to ICT training. Our analysis was guided by the Technology Acceptance Model (TAM).

## Methods

### Conceptual Framework

**Figure 1.**
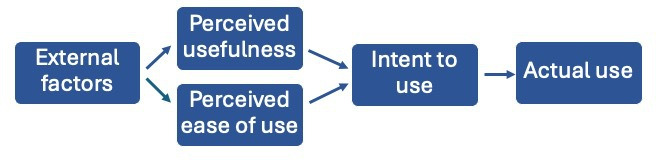
The Technology Acceptance Model.

To explore the anticipated acceptability of a proposed blended learning implementation package, we used the TAM to guide data analysis. TAM is a theoretical model that examines constructs influencing the acceptance or rejection of technologies [26]. The model proposes that acceptability of any technology can be predicted by 2 factors: perceived usefulness and perceived ease of use (Figure 1). Perceived usefulness refers to the belief that a certain technology would increase an individual’s job performance. Perceived ease of use is a belief that the use of the new technology would be effortless [27]. TAM proposes that a populations’ perceptions of usefulness and ease of use of a technology impact the population’s intention to use the technology, which in turn influences the population’s actual usage of the technology. Furthermore, the key constructs of perceived usefulness and perceived ease of use are influenced by external factors, such as pedagogy, digital literacy, infrastructure, and the learning environment. Understanding these external factors is essential for designing a such a package. We used the TAM in this formative sub-study to guide the analysis of the relevant external factors influencing perceived usefulness and perceived ease of use surrounding blended learning in the context of ICT.

### Setting

This study was conducted at health centers in Balaka and Machinga, two districts in Southeastern Malawi. Malawi is a country in Southeastern Africa with an adult HIV prevalence of 8.9% [1]. Although Malawi has met the second two UNAIDS 95-95-95 targets, it has not yet reached the first 95% HIV testing target, with only 88.3% of adults living with HIV aware of their HIV status at the time of the study [1]. Simultaneously, Malawi has one of the lowest rates of qualified HCWs globally with less than 1 medical doctor per 10,000 people [28], resulting in task shifting of HIV services to lay cadres with less formal training [29]. Particularly relevant to the proposed blended learning strategy, is the fact that Malawi has one of the lowest digital literacy levels (having the necessary skills and knowledge to confidently and effectively use technology) globally ranking 131 out of 134 countries by Network Readiness Index [30].

### Study Design and Population

This formative work was conducted to inform a cluster randomized controlled trial designed to examine the implementation, effectiveness, and cost-effectiveness of a blended learning ICT package [23]. Data for this qualitative substudy were generated through IDIs conducted with lay HCWs between November and December 2021. This formative study purposively selected facilities from both districts (Machinga and Balaka) and different levels of care (dispensaries, health centers, and hospitals) for purposes of generalizability. To be eligible for participation, HCWs had to be at least 18 years old, work full-time at one of the health facilities, and be involved in counseling index clients or tracing contact clients. With engagement from facility supervisors, the research team identified up to 2 eligible participants in each facility. These individuals were actively involved in ICT at the time of the study. A research team member visited all the eligible participants in the 14 facilities to give them more details about the study and to seek their consent. Contact details of all consenting participants were shared with one of the research team members who made arrangements with the participants for the date and time of the interviews.

### Data Collection

IDIs were conducted by 3 trained Malawian qualitative interviewers in Chichewa, the local language. All interviews were conducted by 3 female external interviewers. Among them, 1 interviewer had a doctoral degree in social sciences and 2 had bachelor’s degrees in social sciences. The interviewers were trained on techniques of normalization and probing to reduce the risk of socially desirable response bias and had previous training and experience in qualitative data collection and research positions within the organization (postdoctoral fellow, senior research officer, and research officer). Furthermore, interviews were conducted in a private setting at the facilities, with only the interviewers and participants present. Interviews were conducted using a semistructured guide developed for the study through a series of rigorous, iterative discussions among the research team. The questions for this analysis focused on feasibility and acceptability of a blended learning implementation package.

After consenting and before conducting the interview, the research staff introduced themselves and their role, explained the purpose of the interview, and reiterated that they could refuse to answer questions, that all data would be anonymized, and that responses would have no consequences on their employment. Interviewers first asked questions about participants’ views of the standard training. Interviewers then provided participants with a brief description of the planned blended learning approach, explaining that it involved tablet-based asynchronous lessons modeling counseling approaches, as well as interactive synchronous in-person group sessions to practice the skills learned. Finally, they asked about perceived feasibility, probing on potential barriers. Before the start of the IDIs, the interview guides were translated, back translated, and pilot tested with iterative refinement. Interviews were conducted over the phone or face-to-face and lasted approximately an hour long. Repeat interviews were not carried out. Interviewers summarized each IDI immediately after it was conducted. Audio recordings were transcribed verbatim in both Chichewa and translated to English by professional transcribers. Transcriptions were not returned to the participants.

### Data Analysis

The team took a thematic approach to coding [31]. First, the team read the summaries to identify key themes and observed that they were framed by TAM. The research team then developed a qualitative codebook based on a focused examination of the transcripts, debrief reports, and interview summaries. The codebook consisted of both deductive codes from the interview guides and inductive codes that mapped onto TAM constructs. In addition, 2 trained research analysts (TEM and CJM) applied codes to transcript text using the Dedoose (SocioCultural Research Consultants) web application. As a first step, each analyst independently coded the same transcript and compared code applications to ensure agreements in the coding process. The analysts discussed and resolved any coding disparities, updating the codebook as needed to facilitate a shared understanding of all codes. The analysts then met weekly throughout the coding process, which provided space to review code applications and address any challenges. After coding was completed, analysts reviewed the coded text to extract key themes through the identification of patterns, naming and defining themes and refining the identified themes. Analysis was organized by factors that influence the TAM’s constructs: perceived usefulness (what participants perceived as gained or lost by the blended learning implementation package) and perceived ease of use (what participants perceived as facilitators or barriers to acceptance of the blended learning implementation package). The findings were not shared with the participants for their feedback.

### Ethical Considerations

Ethical clearance was provided by the University of North Carolina at Chapel Hill institutional review board (#20‐1810), the Malawi National Health Sciences Research Committee (#20/06/2566) and the Baylor College of Medicine institutional review board (H-48800). Interviewers obtained written informed consent from all participants before starting the IDIs, reminding participants that their participation was voluntary and could be withdrawn at any time. Participants were compensated the equivalent of US $10 per interview. All data were deidentified to protect participant confidentiality.

## Results

### Demographic Summary

A total of 14 facilities participated in the IDIs. Among those, 2 of the facilities were district hospitals, 9 were health centers, and 3 were dispensaries. Furthermore, 9 facilities were in Machinga and 5 were in Balaka. In total, 26 HCWs participated in the IDIs. A total of 7 were female and 19 were male. Furthermore, 16 participants were from Machinga and 10 were from Balaka. There were no participants that refused to participate. All the participants were involved in both screening and counselling index clients and conducting contact tracing and had previous exposure to standard ICT training.

### Themes

**Figure 2.**
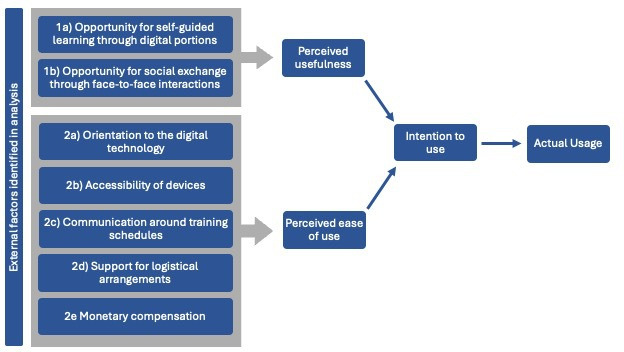
External factors that contribute to perceived usefulness and perceived ease of use.

Our analysis identified two sets of factors influencing perceived usefulness: (1a) opportunity for self-guided learning through digital portions and (1b) opportunity for social exchange through face-to-face interactions. We identified 5 factors influencing perceived ease of use. In addition, 2 factors; (2a) need for orientation to the digital technology and (2b) accessibility of devices were related to perceived ease of use to the technology itself, while 3 factors (2c) communication around training schedules, (2d) support for logistical arrangements, and (2e) monetary compensation were related to perceived ease of use of the training approach (Figure 2). The following sections develop these themes, and Table 1 shows the illustrative quotes.

**Table 1.** Detailed explanations and illustrative quotations for each external factor.

External factors			Quote
**1. Perceived usefulness**			
	**a. Opportunity for self-guided learning**		
		Able to learn better.	…. because I will be doing the things myself, I will be able to understand unlike having someone read to me or stand in front and say that this is this. [P 8021]
		Able to review the training content at their own pace	You can repeat at your own time where you do not understand. [P 8007]
	**b. Opportunity for interaction**		
		Ability to ask questions and seek clarifications.	….the tablet cannot respond to questions that one may have as you do when you have a facilitator. [P 8003] …. with the face-to-face portion if you have other questions maybe of which you failed to ask the tablet, … then you will ask the facilitator and he will. [P 8024]
		Ability to practice and receive feedback.	… face to face, gives us … a chance to be practicing and see which areas we are doing well and areas in which we are lacking and help one another while we are together. [P 8013]
		Interacting with others	… it means we will be able to meet people from different facilities and we will be able to share experiences. [P 8006]
**2. Perceived ease of use**			
	**a. Need for orientation to the digital technology.**		
		Need for orientation on use of tablet.	There can be some people who have never used a tablet and before the training, they should be asked; “have you ever used a tablet?” If the answer is “no,” they should be taught how to use the tablet. [P 8011]
	**b. Accessibility to the digital services**		
		Questions and concerns regarding tablet availability	….if there would be a number of people with few tablets that will be a ‘challenge’ since you will be waiting for your friends to finish then wait for another one. [P 8023] …if the networks are malfunctioning, it will definitely affect your work. [P 8012]
		Technical issues affecting usage and safety that could affect tablet access.	…okay maybe at the time when you are using it [tablet] (…) maybe has run out of power or maybe that thing has a technical fault, [P 8007] ….the tablet can get lost or maybe it fell down and it has broken. [P 8009]
	**c. Communication around training schedules**		
		Engaging in open communication about why certain individuals have been selected to attend tablet training.	People can have thoughts if they can be seeing you working on a tablet and if the supervisor did not say that [name of participant] will be working on a tablet on this day. Your friends can think that you are just playing but if the supervisor can tell them they can know what is going on. [P 8004]
	**d. Logistical arrangements**		
		Location—Preference for the training location	I would love if the training can be conducted in my catchment area and the other people would also want the training to be conducted in their catchment area. So, my opinion is that the training should be conducted at a convenient venue for most of the participants. [P 8006] ….Just because’ maybe ‘you are far from’ your home, ‘you feel’ that you are at a training… [P 8007]
		Time – perception of having the training over the weekend.	So, if the training has happened on a weekend, there’s no problem, it is your free time. [P 8012] …we like using the weekend to…focus on our own things for our lives yeah so maybe many people would be affected if the training is done during the weekend. [P 8007]
	**e. Monetary compensation**		
		Need for allowance.	On the allowances I can say that it would be good if we are given a little something so that we can be assisted. [P 8019] …an allowance is an allowance. It needs to be there. [P 8022]
		Allowances motivate participants to concentrate.	…this motivates people and even the concentration during the training is not compromised. [P 8006] The benefit of receiving allowances is that the money helps you to care for yourself while you are doing the training, it can also help your family at the time when you are away from the family. It will help you to focus on the trainings and your home will not be disturbed due to lack of money. [P 8003]
		Need for equity when giving allowances.	… So, the one working in NGO, because the training is hosted by the NGO, they only receive dinner allowance while those from government they receive dinner and daily subsistence allowance (DSA). [P 8005]
		Compensation is not the sole motivation	I am not very concerned about the allowances because they just motivate us with the allowances…. what is paramount is the new knowledge that that we gain from the training. [P 8002]

### Perceived Usefulness

#### Opportunity for Self-Guided Learning Through Digital Portions

Participants viewed the digital portion of blended learning as an opportunity for self-guided instruction. Some participants expected that a tablet-led training would allow them to learn ICT content better than facilitator-led training. This stemmed from a desire to review the training content at their own time and pace with no disturbances and to repeat difficult sections as needed. Participants expected they would keep the tablets at their facilities for future reference, an appealing feature.

#### Opportunity for Social Exchange Through Face-to-Face Interactions

Participants viewed opportunities to interact with facilitators and colleagues in-person as a central part of learning. Although participants were interested in individual learning, almost all also wished for social interaction during the learning process. Most participants were worried that working on the tablet would be like “talking to oneself” with no feedback mechanisms. They expected they would not get immediate feedback and clarifications. Participants viewed face-to-face sessions as essential for seeking clarification. In addition, participants felt face-to-face learning would allow them to practice and receive feedback. Practicing the skills with a facilitator and colleagues was considered useful because it would help them reinforce content and ultimately facilitate acquisition of knowledge and skills. It was also a time in which participants could receive support from their peers and facilitators on areas they needed to strengthen. In addition, participants considered the sharing of experiences with colleagues as integral.

### Perceived Ease of Use

#### Need for Orientation to the Digital Technology

Orientation to the technology was highlighted as a key factor that would facilitate the ease of use of the blended learning implementation package. Conversely, some participants expressed fear about using a tablet for training without proper orientation due to not being well acquainted with the technology. To this end, participants suggested that they needed orientation on the use of the tablet as well as the applications that would be used during the training since technological skills varied across participants.

#### Accessibility of Devices

Equipment access was a significant factor influencing perceived ease of use. Participants typically did not have personal tablets and assumed a tablet would be provided. Participants had several questions regarding the availability of the tablets within their facility, such as the duration when they would be available and whether they would be assigned to individuals or the facility. In addition, participants identified tablet storage, tablets running out of power, and poor network connectivity as key challenges that would affect tablet access. Participants feared that that the tablets were at risk of being lost at the facility, thus having appropriate tablet storage measures was suggested. Furthermore, participants were concerned that in seasons with frequent power blackouts, they would have challenges to charge the tablets if they ran out of power. Some participants stated that poor mobile network connectivity in some facilities could affect their ability to complete a tablet-based training.

#### Communication Around Training Schedules

Participants emphasized the need for open communication regarding training arrangements. They noted that training attendance can evoke envy among colleagues. To address this, participants suggested ensuring prior communication to all health care workers about the selection process and purpose of training, especially for tablet training, to minimize work disruption and misunderstandings.

#### Support for Logistical Arrangements

Time and location for both portions of the training were highlighted as key logistical considerations that would drive ease of use. Most participants considered having digital training at their place of work or a nearby location acceptable. They also were open to having portions of the training over the weekend or during workdays, with pros and cons for each of these different locations and times. Weekend training was considered desirable by some as it would not disrupt work schedules, but others noted it infringed on their personal commitments. Some participants explained that having the training on-site involved less effort for travel and would not disrupt the work schedule at the facility. However, to some individuals, having the training offsite was important because it created a mindset of higher excitement, motivation, and a sense of purpose for participants. They also noted that participants in offsite training would experience fewer distractions than in a work environment, allowing participants to fully focus on the training content.

#### Monetary Compensation

Provision of a training allowance (ie, monetary compensation) was noted as a contributor to ease of use of a blended training. The norm in the Malawian context is to receive an allowance to cover accommodation and meals when attending a centralized face-to-face training. Participants were asked to share their views regarding such allowances under a decentralized blended learning approach. The general perception was that they would still expect to receive an allowance. In fact, some participants indicated that they preferred offsite trainings, specifically because of the certainty of an allowance. They described how allowances act as a motivating factor that allows people to focus on training content and apply what they learn. In addition, allowances were perceived as important because they support the basic expenses for their household needs without detracting from their family’s financial commitments while they were in the training. Participants preferred to receive a monetary allowance, rather than meals and accommodation, because it provided them with the possibility to save some of the money for other purposes. Participants voiced a desire for allowances to be disbursed equally across organization type, distance travelled, and rank. Although most participants noted allowances as important, many emphasized that this was not their sole ore even primary motivation for training.

## Discussion

This study explored the anticipated acceptability of blended learning among lay HCWs in Malawi. Interest in self-guided learning was a key factor underlying perceived usefulness in digital learning. The desire for interaction was an important consideration for in-person learning. With respect to ease of use, blended learning could be facilitated through orientation, accessibility of digital devices, communication, and consideration of training logistics and compensation.

This study is among the first to examine perceived acceptability of a blended learning implementation package within the context of in-service training among lay HCWs in LMICs. Our study suggests that the use of technology could be acceptable among HCWs in this setting despite low digital literacy, as long as there is sufficient orientation and device access. The study revealed positive perspectives from HCWs on the use of the digital component as it would allow participants to learn the training content better at their own time, place, and pace. Consistently, studies that have used digital technology in tertiary education throughout health professions have reported positive perspectives [32-35], due largely to the ability to review content [16]. We posit that if blended learning proves acceptable in a country with one of the lowest digital literacy levels and many technology barriers, it can also be implemented in areas with higher digital literacy and fewer barriers.

Our findings support previous findings that suggest learners do not want to completely forego human interactions, which they perceive enhance learning [16,17,36-38]. While most participants welcomed the idea of digital ICT training, the lack of interaction was highlighted as a major hindrance to the use of technology alone. Hence, participants preferred to also have a face-to-face portion to resolve their unanswered questions and allow them to practice new skills. The desire for face-to-face encounters has also been highlighted in clinical training programs [32,39]. These findings around interest in both modalities reinforce the value of blended approaches [40] in which participants experience the flexibility of digital self-training and engagement and interactivity in face-to-face sessions [41]. We incorporated these findings into our package by having a set of digital modules focused on reviewing content, followed by face-to-face modules focused on practicing and receiving feedback.

Orientation was perceived as a key factor to facilitate the ease of use of digital technologies, such as tablets in this setting. Participants came from a setting with low technological literacy. According to the World Bank, mobile phone penetration in Malawi was at only at 60% in 2021, suggesting that digital skills are not as ubiquitous as they are in other settings. Therefore, for a blended learning implementation package to be successful, participants need basic navigation skills before initiation [14], as well as technical support to improve ease of use [42]. We incorporated these findings into our package training site supervisors on how to use the blended learning package. The site supervisors, in turn, were empowered to orient staff at their site and support those who experienced technology challenges.

Participants had different preferences regarding the time and location of the training, but they unanimously agreed on the desire for allowances, even with this new training modality. In addition, there was consensus around the importance of equity when distributing allowances. Allowances were felt to serve as motivating factors that allow participants to focus on training content. Such allowances are often provided to motivate participants [43-45]. However, an appeal of the blended learning approach is its potential to expand the skill set of HCWs in a more economical and less disruptive manner. By offering tablet-based training at or near their workplace, HCWs spend less time away from their other duties, and the overall costs could be reduced if no lodging or transportation expenses were incurred. Our study team took these findings under consideration in the development of a blended learning package that is now being tested in a randomized trial. In the trial, to address equity considerations, we offered the training to all lay health workers at each facility and provided a small allowance to recognize participants’ time.

Our study has several limitations and next steps. The study was conducted before HCWs were exposed to blended learning and evaluated anticipated perceptions; actual perceptions may differ. This is something we will evaluate when we implement our trial. In addition, the study was conducted in a few facilities in 2 districts and perceptions may differ in other settings. However, we sampled from a broad range of districts and facility types and achieved saturation. These facilities are not meaningfully different from other facilities in these districts or the country overall, suggesting that our results may generalize broadly in Malawi.

In summary, our formative study showed that a blended learning implementation package may be an acceptable method of training HCWs in the Malawian setting if program developers attend to contextual factors that affect perceived usefulness and ease of use. Although this work offered immediate insights for our planned trial, we recommend additional research to assess acceptability of blended learning approaches in a range of LMIC settings among lay health workers.

## Abbreviations

HCW: health care worker
ICT: index case testing
IDI: in-depth interview
LMIC: low- and middle-income country
TAM: Technology Acceptance Model
UNAIDS: Joint United Nations Programme on HIV and AIDS
WHO: World Health Organization

## References

[1] Malawi Ministry of Health (2022). Malawi final report 2020-2021. https://phia.icap.columbia.edu/malawi-final-report-2020-2021/.

[2] World Health Organization (2016). Guidelines on HIV Self-Testing and Partner Notification: Supplement to Consolidated Guidelines on HIV Testing Services.

[3] Rosenberg NE, Mtande TK, Saidi F (2015). Recruiting male partners for couple HIV testing and counselling in Malawi’s option B+ programme: an unblinded randomised controlled trial. Lancet HIV.

[4] Kahabuka C, Plotkin M, Christensen A (2017). Addressing the First 90: a highly effective partner notification approach reaches previously undiagnosed sexual partners in Tanzania. AIDS Behav.

[5] Tembo TA, Kim MH, Simon KR (2019). Enhancing an HIV index case testing passive referral model through a behavioural skills-building training for healthcare providers: a pre-/post-assessment in Mangochi District, Malawi. J Int AIDS Soc.

[6] Picciano AG (2006). Blended learning: implications for growth and access. OLJ.

[7] Graham CR, Woodfield W, Harrison JB (2013). A framework for institutional adoption and implementation of blended learning in higher education. Internet High Educ.

[8] Sakina R, Kulsum EM, Uyun AS (2020). Integrating technologies in the new normal: a study of blended learning. ijqrm.

[9] Means B, Toyama Y, Murphy R, Baki M (2013). The effectiveness of online and blended learning: a meta-analysis of the empirical literature. Teachers College Record: The Voice of Scholarship in Education.

[10] Karamizadeh Z, Zarifsanayei N, Faghihi AA, Mohammadi H, Habibi M (2012). The study of effectiveness of blended learning approach for medical training courses. Iran Red Crescent Med J.

[11] Singh H (2021). Challenges and Opportunities for the Global Implementation of E-Learning Frameworks.

[12] Skulmowski A, Rey GD (2020). COVID-19 as an accelerator for digitalization at a German university: Establishing hybrid campuses in times of crisis. Hum Behav Emerg Technol.

[13] Finlay MJ, Tinnion DJ, Simpson T (2022). A virtual versus blended learning approach to higher education during the COVID-19 pandemic: The experiences of A sport and exercise science student cohort. J Hosp Leis Sport Tour Educ.

[14] Batubara HS, Riyanda AR, Rahmawati R, Ambiyar A, Samala AD (2022). Implementasi model pembelajaran blended learning di masa pandemi Covid-19: meta-analisis [Article in Indonesian]. basicedu.

[15] Mali D, Lim H (2021). How do students perceive face-to-face/blended learning as a result of the Covid-19 pandemic?. The International Journal of Management Education.

[16] Baber H (2022). Social interaction and effectiveness of the online learning – A moderating role of maintaining social distance during the pandemic COVID-19. AEDS.

[17] Nacaroğlu O, Kızkapan O, Demir H (2025). Middle school students’ motivations and learning competencies in science: mediating role of digital literacy. Psychol Sch.

[18] Islam S, Baharun H, Muali C (2018). To boost students’ motivation and achievement through blended learning. J Phys: Conf Ser.

[19] Ramachandran R, Kaur R, Roy A, Goel P, Deorari AK (2024). Perceived usefulness of a blended learning approach for skills training among medical interns: a pilot study. BMC Med Educ.

[20] World Health Organization (2019). WHO Guideline: Recommendations on Digital Interventions for Health System Strengthening: Web Supplement 2: Summary of Findings and GRADE Tables.

[21] Kenney J, Newcombe E (2011). Adopting a blended learning approach: challenges encountered and lessons learned in an action research study. OLJ.

[22] Tembo TA, Mollan K, Simon K (2024). Does A blended learning implementation package enhance HIV index case testing in Malawi? A protocol for A cluster randomised controlled trial. BMJ Open.

[23] Hamilton AB, Finley EP (2019). Qualitative methods in implementation research: An introduction. Psychiatry Res.

[24] Elsey H, Khanal S, Manandhar S (2015). Understanding implementation and feasibility of tobacco cessation in routine primary care in Nepal: a mixed methods study. Implementation Sci.

[25] Marangunić N, Granić A (2015). Technology acceptance model: a literature review from 1986 to 2013. Univ Access Inf Soc.

[26] Wallace LG, Sheetz SD (2014). The adoption of software measures: A technology acceptance model (TAM) perspective. Information & Management.

[27] (2020). Medical doctors (per 10,000).

[28] Flick RJ, Simon KR, Nyirenda R (2019). The HIV diagnostic assistant: early findings from a novel HIV testing cadre in Malawi. AIDS.

[29] (2023). Network readiness index 2023-malawi.

[30] Clarke V, Braun V, Hayfield N (2015). Qualitative Psychology: A Practical Guide to Research Methods.

[31] Kelly RF, Mihm-Carmichael M, Hammond JA (2021). Students’ engagement in and perceptions of blended learning in a clinical module in a veterinary degree program. J Vet Med Educ.

[32] Bhat S, Madi̇ M (2022). Blended learning in undergraduate dental education. Cumhuriyet Dent J.

[33] Nagaraj C, Yadurappa SB, Anantharaman LT, Ravindranath Y, Shankar N (2021). Effectiveness of blended learning in radiological anatomy for first year undergraduate medical students. Surg Radiol Anat.

[34] Bouarar AC, Mouloudj S, Umar TP, Mouloudj K (2023). Antecedents of physicians’ intentions to engage in digital volunteering work: an extended technology acceptance model (TAM) approach. JICA.

[35] Jiang W (2017). Interdependence of roles, role rotation, and sense of community in an online course. Distance Education.

[36] Wu JH, Tennyson RD, Hsia TL (2010). A study of student satisfaction in A blended e-learning system environment. Comput Educ.

[37] Jusoff K, Khodabandelou R (2009). Preliminary study on the role of social presence in blended learning environment in higher education. IES.

[38] Koch J, Ramjan LM, Everett B, Maceri A, Bell K, Salamonson Y (2020). “Sage on the stage or guide on the side”-Undergraduate nursing students’ experiences and expectations of bioscience tutors in A blended learning curriculum: A qualitative study. J Clin Nurs.

[39] Suwannaphisit S, Anusitviwat C, Tuntarattanapong P, Chuaychoosakoon C (2021). Comparing the effectiveness of blended learning and traditional learning in an orthopedics course. Ann Med Surg (Lond).

[40] Kemp N, Grieve R (2014). Face-to-face or face-to-screen? Undergraduates’ opinions and test performance in classroom vs. online learning. Front Psychol.

[41] Kassably C, Ashaal A, Tahan S, Bouzakhem N, Youssef S, Ghosn F (2024). Identification of factors influencing educators perception of blended learning and triggering adoption decisions. Migrat Lett.

[42] Nkamleu GB, Kamgnia BD (2014). Uses and abuses of per-diems in africa: a political economy of travel allowances.

[43] Vian T, Miller C, Themba Z, Bukuluki P (2013). Perceptions of per diems in the health sector: evidence and implications. Health Policy Plan.

[44] Samb OM, Essombe C, Ridde V (2020). Meeting the challenges posed by per diem in development projects in southern countries: a scoping review. Global Health.

[45] Derntl M, Motschnig-Pitrik R Patterns for blended, person-centered learning: strategy, concepts, experiences, and evaluation.

